# Jejunal Dieulafoy lesion with intraintestinal calcification on computerized tomography: A case report

**DOI:** 10.1097/MD.0000000000026229

**Published:** 2021-06-25

**Authors:** Mudan Wang, Haijun Cao, Jinfeng Dai, Shanshan Chen, Li Xu, Shangao Li

**Affiliations:** a Department of Emergency and Trauma Center; b Department of Gastrointestinal Medicine, The First Affiliated Hospital of Zhejiang Chinese Medical University, Hangzhou, China.

**Keywords:** computed tomography, Dieulafoy lesion, endoscopy, gastrointestinal bleeding, jejunum

## Abstract

**Rationale:**

A Dieulafoy lesion is a rare cause of gastrointestinal (GI) bleeding, especially in the jejunum, and the presence of calcifications on CT might be suspicious of the diagnosis.

**Patient concerns:**

We describe a 72-year-old woman with anemia and melena. Hemoglobin was 6.0 g/dL, and the stools were positive for occult blood (4+). Blood pressure was 116/54 mm Hg. Physical examination showed pale face and pitting edema in both lower limbs. Abdominal computerized tomography showed calcification in the small intestine of the left lower abdomen. Capsule endoscopy showed a blood clot.

**Diagnoses:**

Dieulafoy lesion.

**Interventions:**

Single balloon endoscopy was performed via the oral approach and showed a blood clot on the suspected submucosal tumor of jejunum. A hemostatic clip was placed at the base of the lesion to allow the surgeon to locate it during the operation. Laparoscopy was performed, and the lesion was resected.

**Outcomes:**

The postoperative pathology showed a Dieulafoy lesion. The lower extremity edema subsided. GI bleeding did not recur over 1 year of follow-up, and hemoglobin was 12.2 g/dL. A Dieulafoy lesion is a rare cause of GI bleeding, and it is even rarer in the jejunum.

**Lessons:**

A Dieulafoy lesion does not have special imaging features, but the presence of calcifications in the small intestine on computerized tomography might be suspicious of the diagnosis. When endoscopic treatment is difficult, surgical treatment could be considered.

## 1 Introduction

The Dieulafoy lesion is an uncommon cause of gastrointestinal (GI) bleeding and accounts for 1% to 2% of episodes of acute upper GI bleeding.^[1]^ The lesions are usually present in the stomach and rarely found in the jejunum. Due to the advances in small bowel mucosal imaging, including techniques such as capsule endoscopy and balloon-assisted endoscopy, the rate of diagnosis of small bowel lesions has markedly increased. Nevertheless, Dieulafoy lesion in the small bowel is difficult to diagnose and is likely an underdiagnosed cause of occult GI bleeding.^[2,3]^ Calcification may be an external manifestation of a certain tumor or inflammatory lesion. However, it is rare for calcification to be found in hemorrhage foci. We report a case of Dieulafoy lesion in the jejunum, and the presence of calcifications on computerized tomography (CT) might be suspicious of the diagnosis.

## 2 Case presentation

A 72-year-old woman was admitted to the First Affiliated Hospital of Zhejiang Chinese Medical University on January 2, 2018 due to fatigue and melena for more than 10 days. She had dizziness and chest tightness, but no abdominal pain, nausea, vomiting, or change in weight. Standard care is performed, so ethical approval is not applicable in this study. Written informed consent was obtained from the patient.

She had a history of hypertension for more than 10 years, but no history of anticoagulants, nonsteroidal anti-inflammatory drugs, or alcohol use.

**Figure 1:**
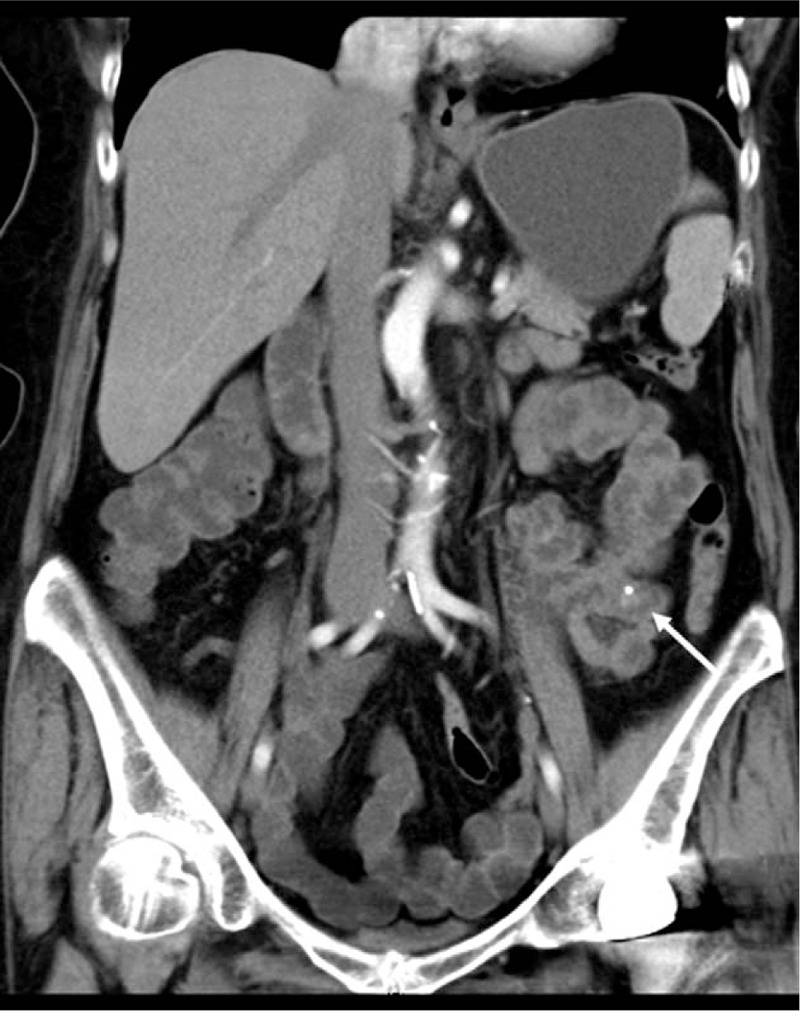


**Figure 2:**
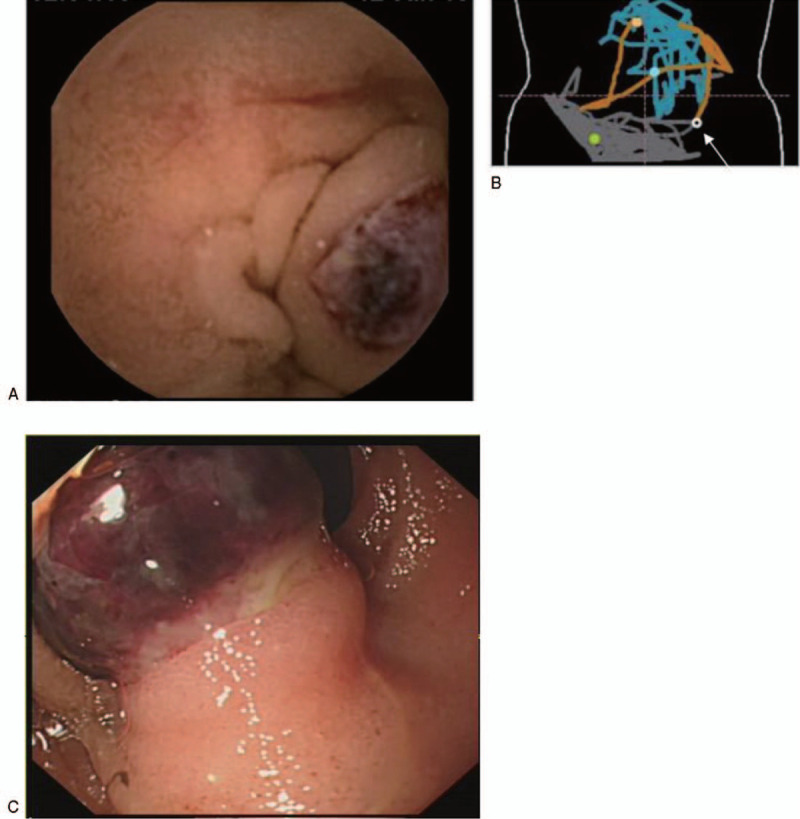


Physical examination showed pale face and pitting edema in both lower limbs. Hemodynamics were stable, with a blood pressure of 116/54 mm Hg. Hemoglobin was 6.0 g/dL, and the stools were positive for occult blood (4+). Gastroscopy revealed anemia-induced gastric mucosal changes, but no bleeding lesions were found during gastroscopy and colonoscopy to the terminal ileum. CT showed calcification in the small intestine of the left lower abdomen (Fig. 1). CT angiography of the superior mesenteric artery did not show significant abnormality. A capsule endoscopy (Given Imaging, Israel) examination revealed bleeding in the upper jejunum, with a blood clot attached (Fig. 2A, B).

### 2.1 Final diagnosis

GI bleeding.

### 2.2 Treatment

**Figure 3:**
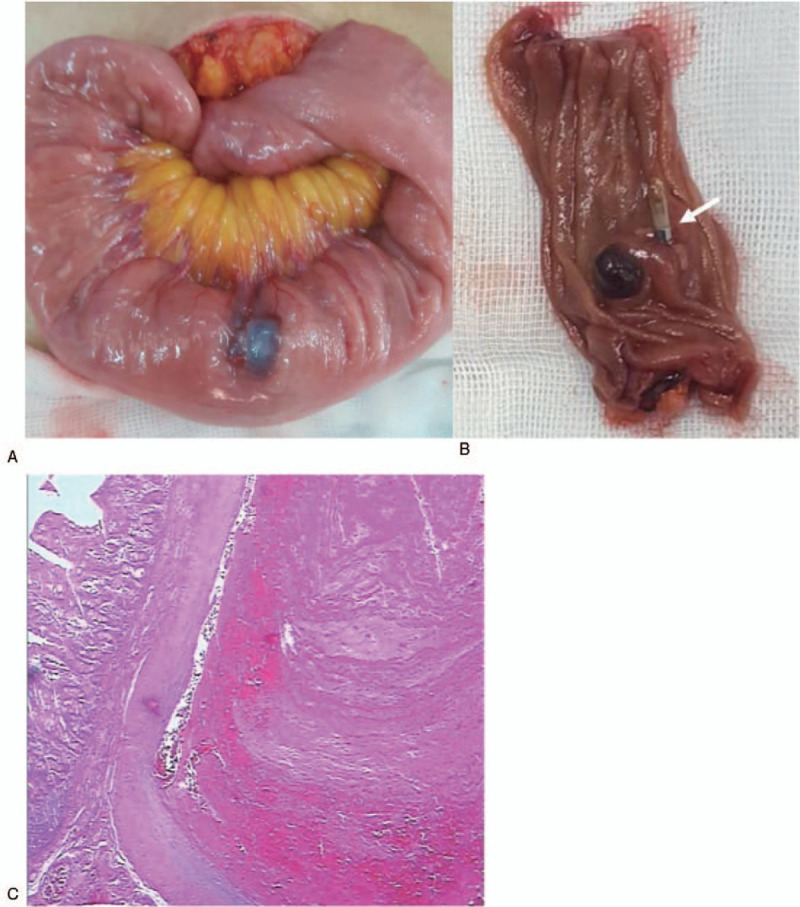


The patient was infused with 2 units of red blood cells and sucrose iron for improving anemia, and octreotide was used for contracting the splanchnic vessels. Single balloon enteroscopy (SIF-Q260, Olympus) showed a submucosal tumor-like bulge with a diameter of 2.0 cm at 70 cm from the Trietz ligament, with a blood clot attached on the surface (Fig. 2C). A hemostatic clip was placed at the base of the lesion to allow the surgeon to locate the lesion during the operation. Laparoscopy was performed after discussion with the patient, and the lesion was found in the left lower abdominal jejunum, as expected. Then the incision was lengthened to 5 cm, and open surgery was done to resect the lesion. A dark purple mass was observed at about 70 cm from the Trietz ligament (Fig. 3A), about 2.0 cm in diameter, with tough texture and smooth surface. The hemostatic clip was found (Fig. 3B). A short segment jejunum excision and an end-to-end anastomosis were performed. Histopathological examination of the resected tissues revealed a pseudotumor with a large submucosal dilated artery (Fig. 3C). Vessel wall thickening with degeneration, thrombosis, and interstitial calcification were observed. The histological characteristics were consistent with a Dieulafoy lesion.

### 2.3 Outcome and follow-up

The patient recovered well after the operation. One week later, hemoglobin was 9.3 g/dL, and lower extremity edema gradually subsided. GI bleeding did not recur over 1 year of follow-up, and hemoglobin was 12.2 g/dL on December 13, 2019.

## 3 Discussion

Dieulafoy lesions were first described by Gallard as “miliary aneurysm” in 1884 and were later accurately described by the French surgeon Dieulafoy in 1896.^[4]^ It is currently accepted that the pathological feature of Dieulafoy lesions is the presence of abnormal large arteries under the mucosa and is a rare cause of GI bleeding. Most Dieulafoy lesions (>80%) were described in the proximal stomach 6 to 10 cm from the gastroesophageal junction, and jejunum Dieulafoy lesions are very rare. Lipka et al^[5]^ considered that jejunum Dieulafoy lesions accounted for 2.6% of all Dieulafoy lesions. They reported their own case and reviewed the literature to conclude that the main position of Dieulafoy lesions in the small bowel was the jejunum.

The causes of Dieulafoy lesions include non-steroidal anti-inflammatory drugs, anticoagulants, alcoholism, stress, and heart and lung failure. The currently recognized mechanism is that bleeding originates from arteries with abnormally large diameters under the mucosa. The superficial mucosa of the blood vessel is eroded due to various reasons, leading to the exposure of the blood vessel and rupture with bleeding.

Dieulafoy lesions in the jejunum can cause repeated massive GI bleeding and are not easily diagnosed by conventional methods. It is often necessary to perform technetium-labeled red blood cell scanning or digital subtraction angiography (DSA), but it may be missed due to a transient stop of bleeding during the examination. Capsule endoscopy and/or balloon-assisted endoscopy have excellent capabilities in detecting lesions in the small intestine.^[6,7]^ Meanwhile, calcification may be an external manifestation of some diseases, such as papillary thyroid carcinoma and necrotic adipose tissue and so on.^[8,9]^ In addition, calcification occurred in gastric adenocarcinoma, and the cause is related to the parathyroid hormone secreted by adenocarcinoma.^[10,11]^ However, up to now, there are few reports about calcification associated with GI bleeding. It is worth noting that calcification was found in the gastric Dieulafoy lesion in a renal transplantation patient with visceral calcification, which was reported by Shapiro et al.^[12]^ In this case, CT first identified a clear calcification in the jejunum. Capsule endoscopy was then performed to locate the lesion, and single-balloon enteroscopy was used to determine the characteristic of the lesion. During enteroscopy, the lesion was observed to be similar to a ruptured submucosal tumor with bleeding. This is similar to the case reported by Jeong et al.^[13]^ Dieulafoy lesions do not have specific imaging features.^[14]^ We did not pay much attention to the CT image at first, until we compared it with the real-time location of the capsule endoscopy. We were surprised to find that the location of the calcified lesion in the small intestine on CT and capsule endoscopy was exactly the same. In addition, postoperative pathology confirmed the presence of interstitial calcification. The calcification may be caused by blood clot formation and organization. This finding suggests that for patients with unexplained GI hemorrhage, the presence of calcification on CT images could suggest the possibility of intestinal Dieulafoy lesions.

The jejunum Dieulafoy lesions can be treated with endoscopic treatment such as electrocoagulation, argon plasma coagulation, injection sclerosis or ligation, and DSA intervention.^[15,16]^ Surgery was once considered a first-line treatment for Dieulafoy disease. Currently, about 5% of patients cannot be diagnosed and treated with endoscopic or DSA, and surgical treatment will be chosen.^[14]^ Surgery might reveal complex occult lesions.^[17]^ In this case, the patient showed mucosal tumor-like bulges and thick and big blood vessels by endoscopy, which was deemed not suitable for endoscopic treatment. There was no active bleeding after admission, so DSA treatment was also unsuitable. Therefore, surgical treatment was performed.

A Dieulafoy lesion is a rare cause of GI bleeding, and it is even rarer in the jejunum. A Dieulafoy lesion does not have specific imaging features, but the presence of calcifications in the small intestine on CT might be suspicious of the diagnosis. When endoscopic treatment is difficult, surgical treatment could be considered.

## Author contributions

**Conceptualization:** Mudan Wang.

**Data curation:** Haijun Cao, Jinfeng Dai, Shanshan Chen, Li Xu.

**Formal analysis:** Mudan Wang.

**Project administration:** Haijun Cao.

**Resources:** Shangao Li.

**Writing – original draft:** Mudan Wang, Haijun Cao, Jinfeng Dai, Shanshan Chen, Li Xu, Shangao Li.

**Writing – review & editing:** Mudan Wang, Haijun Cao, Jinfeng Dai, Shanshan Chen, Li Xu, Shangao Li.
